# Patients with healed diabetic foot ulcer represent a cohort at highest risk for future fatal events

**DOI:** 10.1038/s41598-019-46961-8

**Published:** 2019-07-17

**Authors:** Julia K. Mader, Waltraud Haas, Felix Aberer, Beate Boulgaropoulos, Petra Baumann, Marlene Pandis, Karl Horvath, Faisal Aziz, Gerd Köhler, Thomas R. Pieber, Johannes Plank, Harald Sourij

**Affiliations:** 1 Division of Endocrinology and Diabetology, Department of Internal Medicine, Medical University of Graz, Auenbruggerplatz 15, A-8036 Graz, Austria; 2 Joanneum Research Forschungsgesellschaft m.b.H, HEALTH - Institute for Biomedicine and Health Sciences, Neue Stiftingtalstrasse 2, A-8010 Graz, Austria; 3 Institute of General Practice and Evidence-based Health Services Research, Medical University of Graz, Auenbruggerplatz 20, A-8036 Graz, Austria; 4 Center for Biomarker Research in Medicine (CBmed), Stiftingtalstrasse 5, 8010 Graz, Austria; 5 Division of Gastroenterology and Hepatology, Department of Internal Medicine, Medical University of Graz, Auenbruggerplatz 15, A-8036 Graz, Austria

**Keywords:** Atherosclerosis, Diabetes complications

## Abstract

Patients with previous diabetic foot ulcer are prone to re-ulceration and (re)amputation, to various comorbidities, have significantly impaired quality of life and increased mortality. We aimed to evaluate the risk of foot related complications and mortality in a high-risk population of patients with healed diabetic foot syndrome over a decade. 91 patients with recently healed diabetic foot ulcer were invited for follow-up at 1, 6 and 11 years after inclusion. Patient characteristics at inclusion were: 40 women, 65 ± 11 years, diabetes type 1 (n = 6) or 2 (n = 85), BMI 28.5 ± 4.4 kg/m^2^, and HbA1c 68 ± 17 mmol/mol. Comorbidities included neuropathy (n = 91), peripheral artery disease (PAD), history of minor (n = 25) or major (n = 5, 5.5%) amputation, nephropathy (n = 40) and retinopathy (n = 53). Ulceration recurred in 71 (65%) patients, time to first recurrence was 1.8 ± 2.4 years (mean ± SD). 21 patients had to undergo (re)amputation (minor n = 19, major n = 2), time to amputation was 3.6 ± 1.9 years. Over time, 3 further major amputations were required in patients with an initial minor amputation. Thirty-three (36%) of the initially included patients completed the follow-up period of 11.0 ± 0.6 years. 58 patients (64%) died during the observational period, time to death was 5 ± 3 years in this group. We found overall high mortality of 64% throughout the follow-up period of 11 years in high-risk patients with healed diabetic foot syndrome. Presence of PAD, prior amputation and nephropathy as well as poor glycemic control were significantly predictive for death.

## Introduction

Diabetic foot syndrome (DFS) is a late complication in both patients with type 1 and type 2 diabetes mellitus with a prevalence of 4 to 10%. The lifetime risk of patients with diabetes mellitus to develop DFS is as high as 25%^1–3^. Patients with DFS are two to three times more likely to die than patients without DFS^4–8^.

Patients with DFS are predisposed to various comorbidities, such as peripheral artery disease (PAD), cardiovascular disease (CVD), neuropathy, retinopathy, and nephropathy, and have significantly impaired quality of life^3,9–12^. Wound healing of diabetic ulcers is often a long process requiring substantial resources of health care systems^13^. Even after accomplished wound healing, reulcerations occur frequently and commonly lead to minor or major amputation of lower extremities^14,15^.

Several studies to evaluate outcomes in patients with DFS exist^4,5,14–18^, but data on the outcome of patients with healed diabetic foot ulcer are limited. We therefore aimed to investigate the risk of foot related complications, comorbidities and mortality in a high-risk population of patients with healed DFS over a time period of 11 years.

## Patients and Methods

### Study design and patients

Between May 2000 and September 2001, patients with type 1 and type 2 diabetes and recently healed diabetic foot ulcer who presented at the diabetes foot clinic of the Medical University of Graz were invited to participate in the study. Two patients did not consent for personal reasons; 91 were consecutively included in the study. The population of 91 patients was initially randomized to monthly chiropodist care vs. standard foot care program for 12 months. Thereafter routine care was continued in both groups. Patients were followed up 1, 6 and 11 years after inclusion or until death. There was no drop-out or patient lost to follow-up. The following assessments were performed during all follow-up visits: medical history included type, duration and treatment of diabetes, macro- and microvascular comorbidities, amputations of lower limbs and smoker status. Physical examination of the feet included the assessment of peripheral neuropathy, peripheral arterial disease (PAD), recent (re)amputations and (re)ulcerations. Laboratory assessments included HbA1c, creatinine and microalbuminuria. Screening for diabetic retinopathy was performed by an ophthalmologist and data from patient letters were transcribed. The methods and definitions are described in detail elsewhere^17^. Initial visit and follow-up visits were performed by a diabetes specialist and a diabetes nurse. Between study visits regular diabetes or diabetic foot related care was provided to the patients either at Medical University of Graz, at other clinics or at the family doctor as preferred by the patients. Intervals of these appointment was at the discretion of the treating physician.

The study was approved by ethics committee of Medical University of Graz and performed in accordance with the principles of Good Clinical Practice and the Declaration of Helsinki. All participants gave written informed consent prior to any study related activities. The study was retrospectively registered at the German Clinical Trials Register (DRKS00015224).

### Statistical analysis

We performed the statistical analysis in SAS 9.2 and STATA 15.1. In all variables, data with the response ‘unknown’ were treated as missing.

We tabulated descriptive statistics as mean ± standard deviation (SD) if not indicated otherwise. We conducted logistic regression analysis to identify significant predictors of amputation and mortality. We used stepwise regression backward selection method and ‘gvselect’ program of stata to select significant predictors. The results of multiple logistic regression were reported in terms of adjusted odds ratios (AOR) with corresponding 95% confidence intervals (CI) and *p*-values. We excluded ‘type of diabetes’, ‘stroke’. ‘myocardial infarction’, and ‘body mass index (kg/m^2^)’ from the final logistic regression model because of high collinearity between these variables. We generated Kaplan-Meier curves for ‘gender’ and other significant predictors to assess time to amputation and mortality, respectively. We did not adjust for multiple comparisons.

### Prior presentation of data

Parts of this study were presented at the American Diabetes Association’s 75^th^ Scientific Session, Boston, 5–8 June 2015 and the 51^st^ Annual Meeting of the European Association of for the Study of Diabetes, Stockholm, Sweden, 13–18 September 2015.

## Results

Patient characteristics at baseline are indicated in Table 1. During the follow-up period of 11.0 ± 0.6 years, ulceration recurred in 71 patients of the initially included 91 patients. Time to first recurrence was 1.8 ± 2.4 years. Subsequent ulcers after initial reulceration occurred in 35 cases on the same foot and in 37 cases in both feet during the observation period. 21 patients had to undergo (re)amputation during follow-up (minor n = 19, major n = 2), time to amputation was 3.6 ± 1.9 years. Over the follow-up period, 3 consecutive major amputations were required in patients with an initial minor amputation. 58 patients (63.7%) died during the observational period; mean time to death was 5 ± 3 years in this group. Causes of death were cardiovascular (62.1%), infectious (20.7%), malignant (6.9%) or renal (1.7%) disease. Two of the fatal infections were due to DFS. One (1.7%) of the 58 deceased patients committed suicide. Cause of death was unknown for 6.9% of the cohort.

**Table 1:** Baseline characteristics of the study cohort.

n = 91	Clinical characteristics
Age (years)	65 ± 11 years
Female gender	40 (44%)
BMI	28.5 ± 4.4 kg/m^2^
HbA1c	68 ± 17 mmol/mol
Type 1 diabetes	n = 6 (6.6%)
Type 2 diabetes	n = 85 (93.4%)
Clinical signs of neuropathy	n = 91 (100%)
PAD	n = 42 (46.2%)
History of minor amputation	n = 25 (27.5%)
History of major amputation	n = 5 (5.5%)
Nephropathy	n = 40 (44%)
Retinopathy	n = 53 (58.2%)

Baseline characteristics of the study population. Data are mean and standard deviation or number (percentage). BMI – Body Mass Index; PAD – Peripheral Artery Disease.

### Predictors for mortality

In the regression model for death with the predictors PAD, previous amputations, HbA1c, nephropathy as well as the control variable age were significant predictors for death. The odds ratios, confidence intervals (CI), and *p*-values for all tested variables are indicated in Table 2.

**Table 2:** Predictors of mortality and amputation in patients with diabetic foot ulcer (n = 91).

Variable	AOR	95% CI	*P*-Value
	Predictors of mortality		
Age – years	1.08	1.02–1.16	**0.015**
HbA1c ≤58 mmol/mol	9.90	1.79–54.93	**0.009**
Nephropathy	14.05	2.12–93.14	**0.006**
Retinopathy	0.16	0.02–1.08	0.059
Peripheral Artery Disease	5.90	1.37–25.33	**0.017**
Previous amputation	6.42	1.18–34.81	**0.031**
	**Predictors of amputation**		
Age – years	0.93	0.88–0.99	**0.028**
HbA1c ≤58 mmol/mol	4.18	0.79–22.01	0.091
Creatinine – mg/dl	0.15	0.02–1.14	0.067
Insulin therapy	0.31	0.05–1.85	0.197
Peripheral Artery Disease	2.56	0.62–10.62	0.193
Coronary Artery Disease	3.68	0.67–24.28	0.176
Nephropathy	0.20	0.05–0.86	**0.031**
Retinopathy	7.08	1.32–37.91	**0.022**

AOR: Adjusted odds ratio, CI: Confidence Interval, HbA1c: Glycated Hemoglobin.

### Predictors for amputation

The regression model for amputation yielded significant results for the predictor retinopathy and the control variable age. The other factors, including gender, insulin therapy, PAD, CVD, smoking, nephropathy, BMI and HbA1c > 58 mmol/mol were not associated with an increased risk of amputation. Initial treatment allocation (monthly chiropodist vs. standard care) also did not affect amputation risk. The odds ratios, confidence intervals, and *p*-values for all tested variables are indicated in Table 2. The presence of nephropathy was associated a lower risk for amputations.

### Factors associated with survival and amputation

**Figure 1:**
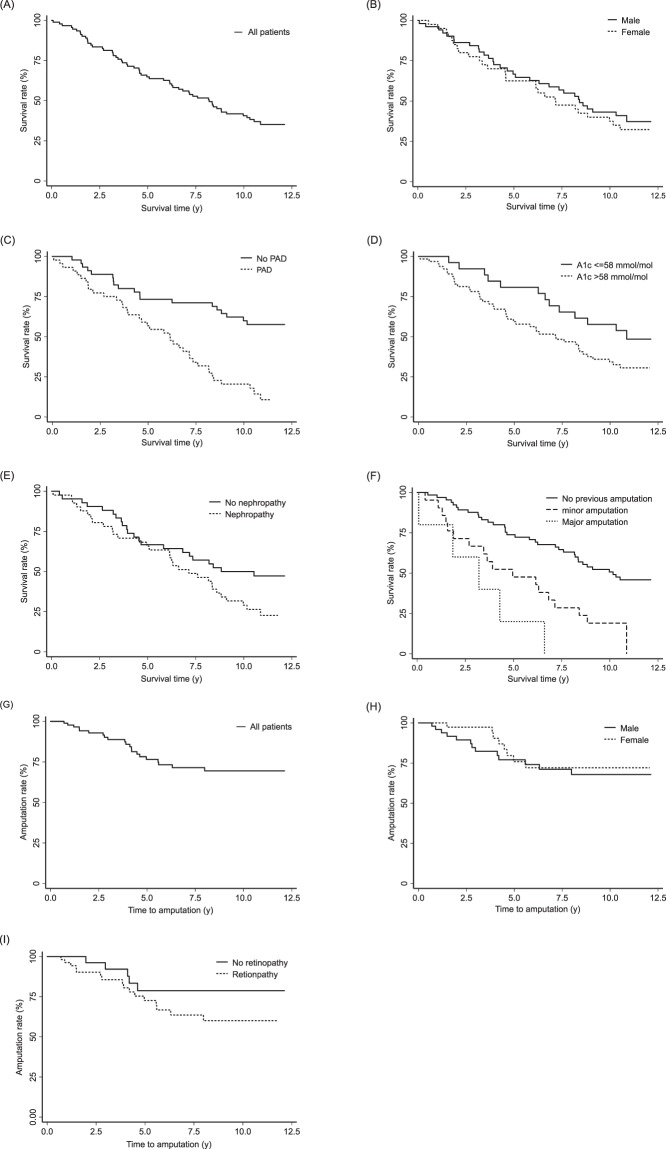
Kaplan Meier Curves for Survival and Amputation. (**A**) Overall survival, (**B**) Survival by gender (female patients - solid line, male patients - dashed line), (**C**) Survival by peripheral artery disease (PAD) status (no PAD - solid line, PAD - dashed line), (**D**) Survival by glycemic control (HbA1c < = 58 mmol/mol (solid line) vs. >58 mmol/mol (dashed line)), (**E**) Survival by nephropathy status (no nephropathy - solid line, nephropathy - dashed line), (**F**) Survival by previous amputation status (No previous amputation - solid line, minor amputation - dashed line, major amputation - dotted line), (**G**) Overall amputation rates (**H**) Amputation by gender (female patients - solid line, male patients - dashed line), (**I**) Amputation by retinopathy status (no retinopathy - solid line, retinopathy - dashed line).

Figure 1 displays the Kaplan-Meier curves for survival and amputation. Overall survival probabilities and survival probabilities with respect to gender, peripheral artery disease (PAD) status, nephropathy and HbA1c were assessed. Additionally, overall risk of amputation and risk of amputation in relation to gender and retinopathy status were investigated. Survival probability for all patients after 11 years of follow-up was 36%, highly irrespective of gender (females: 32.5%, males: 39.2%; see Fig. 1A,B). In contrast, PAD status significantly influenced the survival probability of the patients. Patients without PAD had a survival probability after 11 years of follow-up of about 57.8%, whereas survival probability was only 13.6% for patients with PAD (Fig. 1C). Good glycemic control at inclusion obviously had a legacy effect at 11 years of follow-up: patients with an initial A1c value ≤ 58 mmol/mol had a survival probability of 55%, whereas survival probability for patients with an A1c value > 58 mmol/mol ( > 7.5%) was only 26% (Fig. 1D). The same impact on survival probability was seen for presence of nephropathy (48% for patients without, and about 24% for patients with nephropathy; Fig. 1E).

Overall amputation risk was 23% at 11 years of follow-up; gender was not associated with risk for amputation (females: 20%, males 26%; Fig. 1F,G). Presence of retinopathy increased the risk for future amputations: 30% for patients with retinopathy vs. only 17% for patients without retinopathy (Fig. 1H).

## Discussion

The present study followed a cohort of patients with diabetes mellitus and recently healed diabetic foot syndrome for a period of 11 years. Our main focus was to investigate mortality and amputation rates and predisposing factors in this high-risk population. In our population 63.7% died during the observational period, with cardiovascular events (62.1%) being the main cause of death.

Long-term outcomes after ulceration in diabetic patients have been investigated in several studies^5,16,18,19^ Iversen *et al*. have observed mortality rates of 49% over a ten-year follow-up period and also in this population events were the main cause of death also in this population (48.7%)^5^. Apelqvist *et al*. have reported survival rates of 92%, 73% and 58% for patients with recently healed DFS at 1, 3 and 5 years of observation^14^. This is in rough agreement with the resulting survival rates of about 95%, 80% and 65% after 1, 3 and 5 years in our investigation.

Iversen *et al*. have concluded that older age, male sex, lower education, smoking and larger waist circumference were predictive factors for death in patients with DFS^5^, whereas Morbach *et al*. have reported higher age, male gender, chronic kidney disease including necessity of dialysis and PAD to be predictive for death^16^. We investigated the relation of mortality risk and predisposing factors and found that presence of PAD, prior amputations and nephropathy as well as poor glycemic control status significantly predicted fatal events over a period of 11 years. We further investigated predictors for future amputations and identified the presence of retinopathy and age as significant predictors. Interestingly, nephropathy was inversely associated with future risk for amputations, however, this seems to be due to the competing risk of death, an outcome that is significantly increased by the presence of nephropathy.

Individuals with diabetes mellitus have a two-fold higher risk of all-cause and CVD mortality than individuals without diabetes^20^. Our data indicate annual mortality rates of approximately 6% in patients with diabetic foot syndrome and PAD. This rate is considerably higher than mortality rates reported in recent cardiovascular outcome trials such as EMPA-REG, EXSCEL, LEADER or SUSTAIN. These trials have included mainly subjects with established coronary artery disease and observed annual mortality rates were approximately 2% in such patients considered to be at high-risk for cardiovascular events or death^21–24^. However, the population of patients with diabetes and PAD that is at even higher mortality risk is rather underrepresented in current cardiovascular outcome trials so far. Future trials should be designed to better represent this high-risk population and investigate whether they might also benefit from treatment that mitigates cardiovascular risk.

Moreover patients with PAD experience less intensive risk factor management than patients with CVD which might attribute to higher mortality rates in this population^25^.

We found overall high mortality (64%) throughout the follow-up period of 11 years in patients with healed diabetic foot syndrome. Presence of PAD, prior amputations, nephropathy and poor glycemic control were significantly predictive for death.

## Data Availability

The datasets analysed during the current study are available from the corresponding author on reasonable request.
